# Highly Efficient Processing of Multi-photon States

**DOI:** 10.1038/srep12792

**Published:** 2015-08-06

**Authors:** Qing Lin, Bing He

**Affiliations:** 1 Fujian Provincial Key Laboratory of Light Propagation and Transformation, College of Information Science and Engineering, Huaqiao University, Xiamen 361021, China; 2 Department of Physics, University of Arkansas, Fayetteville, AR 72701, USA

## Abstract

How to implement multi-qubit gates is an important problem in quantum information processing. Based on cross phase modulation, we present an approach to realizing a family of multi-qubit gates that deterministically operate on single photons as the qubits. A general *n*-qubit unitary operation is a typical example of these gates. The approach greatly relax the requirement on the resources, such as the ancilla photons and coherent beams, as well as the number of operations on the qubits. The improvement in this framework may facilitate large scale quantum information processing.

Recently the research on quantum information processing has been approaching the stage of large number of qubits, and numerous exciting developments in this trend have been reported. In 2009, Monz *et al.* reported the creation of 14-qubit entanglement with trapped ions^1^. Later in 2011 and 2012, Huang *et al.*^2^ and Yao *et al.*^3^ reported the generation of eight-photon entanglement, respectively. How to efficiently process the ever increasing number of qubits becomes a prominent problem. Usually people follows the practice of classical computation to decompose a circuit into CNOT gates as the basic two-qubit gate and single-qubit gates. This CNOT-based approach has been adopted in many research works, and its target is to decompose a quantum circuit into as few CNOT gates as possible^4^,^5^,^6^,^7^,^8^,^9^,^10^,^11^,^12^,^13^,^14^. For a two-qubit unitary operation, at least three CNOT gates should be required^9^,^10^; for the well-known *n*-qubit Toffoli gates, 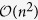 CNOT gates are necessary^5^. Meanwhile, 2^*n*−1^ CNOT gates should be used to implement a general (*n *− 1)-control-1 gate^11^. The theoretical lower bound for a general *n*-qubit unitary operation is 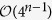 CNOT gates^12^, but the actual number should be up to 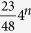 14.

As early as in 2001, Knill, Laflamme and Milburn proposed the architecture of optical quantum computation, based on the prepared multi-photon entangled states as the ancilla^15^. In the same year, Pittman *et al.* presented a scheme to realize CNOT gate based only on linear optics^16^. The CNOT gate has been experimentally demonstrated with linear optics^17^,^18^,^19^,^20^, as well as another two-qubit photonic gate, c-phase gate^21^,^22^. Moreover, numerous multi-qubit photonic gates, e.g. the well known Fredkin and Toffoli gate, were proposed^23^,^24^,^25^,^26^ and implemented^27^ with linear optics.

Though the linear optical realization of photonic quantum circuits is possible, due to the probabilistic nature in operation, its efficiency will not be so high when the processed photon number becomes large. A significant improvement is using weak cross-Kerr nonlinearity, so that the photonic gate operations could be made deterministic. The first application of weak cross-phase modulation (XPM) is the parity check^28^. Afterwards, this technique was adopted to an implementation proposal of deterministic CNOT gate^29^. Following this line of research, many applications of weak Kerr nonlinearity in quantum information processing have been proposed in recent years^30^,^31^,^32^,^33^,^34^,^35^,^36^,^37^,^38^,^39^,^40^,^41^,^42^,^43^,^44^,^45^,^46^,^47^,^48^,^49^,^50^,^51^,^52^. Moreover, photon loss and decoherence effects in weak Kerr nonlinearity were studied from different perspectives^53^,^54^,^55^,^56^,^57^. Going back to quantum computation, the circuit construction in most of previous works belongs to the CNOT-based scenario, i.e. CNOT gate is the elementary gate for constructing any quantum circuit. An alternative route to quantum computation is based on a different type of basic logic gates, c-path and merging gate. The first design of c-path gate based on weak XPM was introduced in 2009^58^, and later the design was simplified and developed to a deterministic one^59^,^60^,^61^. This c-path-merging approach has been experimentally demonstrated with linear optical elements^64^, and the essential idea of c-path and merging gate has also been utilized in other experiments^65^,^66^,^67^. These two universal elementary logic gates can efficiently realize various controlled logic gates such as Fredkin and Toffoli gate. Especially the construction with c-path and merging gates can reduce the complexity of a Toffoli gate from polynomial 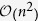 to linear^59^,^60^.

In this paper, based on the improved designs for c-path and merging gate, we will develop the c-path-merging approach to realize various multi-qubit controlled unitary operations and the general *n*-qubit unitary operation. Compared with the CNOT-based approach, various controlled unitary operations can be implemented more efficiently with less resources and less operations. We will show that, for the realization of a general (*n *− 1)-control-1 unitary operation, the required resources, e.g. ancilla coherent states, ancilla single photons, can be reduced from exponential to linear, providing an optimization of such unitary operation. Furthermore, two approaches for realizing a general *n*-qubit unitary operation are proposed.

The rest of the paper is organized as follows. We firstly present the improved optical realization of the two element gates, c-path and merging gate. These element gates will be used to construct various multi-control gates and the general unitary operation in next part. Afterward, we will discuss the complexity of our approach and compare it with the CNOT-based approach. Then the paper is concluded with the final part.

## Element Logic Gates

The operations described below are performed by two element gates, c-path and merging gate^58^,^59^,^60^. Here, for the purpose of clarity, we will first describe a special example of c-path gate and then develop it to more control and target modes. After that we will improve the original merging gate by dispensing with the ancilla single photon in its original design, and also show that it can be generalized to more spatial modes. Compared with the former works^58^,^59^,^60^,^61^, the primary advantage in the current approach is that no ancilla photon is necessary to any circuit, no matter how complicated it could be.

## C-path Gate

**Figure 1:**
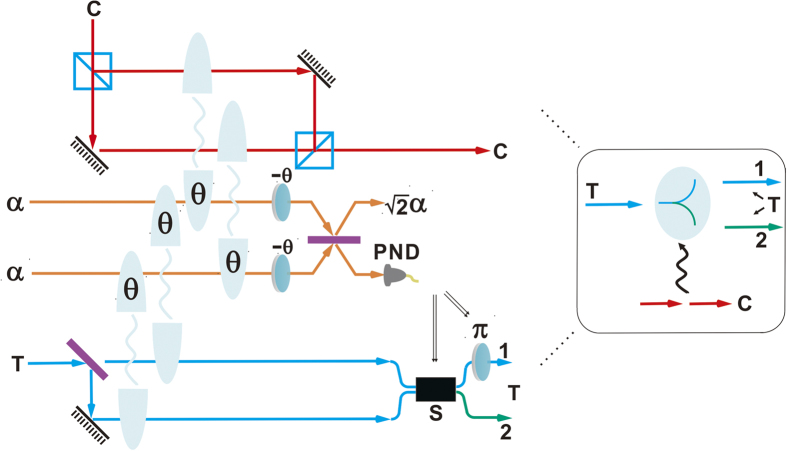
Schematic design of an example of c-path gate. The control photon and target photon contain only one spatial modes. Firstly, the control photon goes through a PBS, and the target photon through a 50:50 BS. Next, the spatial modes interact with the qubus beam as indicated. The operation steps in the following order—phase shift −*θ*, the two coherent states interference, and the detection of the first coherent-state component by a photon number-resolving detector (PND) for controlling the switch and phase shift *π*—implements this c-path gate.

This element gate encodes the bit information of a control qubit into the spatial modes of the target qubit. In Fig. 1 the realization of an example of c-path gate is shown. Here we adopt the definitions 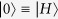 and 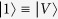, where H and V represent the two polarizations of a single photon, respectively. The input state is as follows,


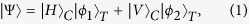


where the states |*ϕ*_1(2)_〉 are in arbitrary forms (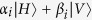, with 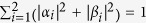. At firstly, let the control photon transmit through a polarized beamsplitter (PBS) and the target photon through a 50:50 beamsplitter (BS). The target photon will be separated into 2 spatial modes (1, 2). Secondly, one introduces two coherent states 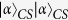 (qubus beams) and let them interact with the input two single photons through XPM as shown in Fig. 1. Then the input state will evolve to the following state


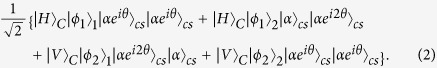


Here we assume the XPM between single-mode coherent state and single-mode one photon state, which is valid under the conditions specified in^79^,^80^. After that, a phase shifter of −*θ* is, respectively, applied to two qubus beams, followed by the transformation 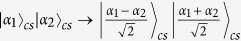 with one more 50:50 BS on the coherent state components. The state of the total system will be therefore transformed to


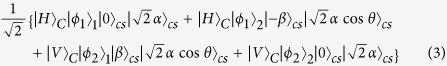


where 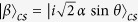 and 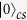 denotes the coherent vacuum state. To obtain the desired state, we need a photon number-resolving detector (PND) to measure the first qubus beam (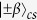), and then separate the first and fourth components from the second and third components in the above state. The photon number resolution, denoted by the projection 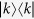, is realized in an indirect way through coherent state comparison; see the first part of Supplementary Material for details about the PND module. If the projection result is *k* = 0, we will obtain the target state


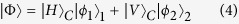


where the spatial modes 1, 2 of the target photon depend on the polarizations 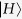, 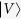 of the control photon, respectively. If 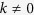, on the other hand, there will be the output


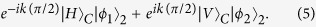


Since the exact photon number *k* is known, it is possible to removed the unnecessary phase shifts *e*^−*ik(π*/2)^ and *e*^*ik*(*π*/2)^ by a conditional phase shift −*kπ* applied on the upper spatial modes, based on the classically feed-forwarded measurement result *k*. Finally, implementing a swapping between the upper and lower spatial modes transforms the above state to the desired one 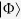.

After implementing the c-path gate, the target photon will be separated into two spatial modes, which depend on the polarizations of the control photon. Then a more general c-path gate realizing multiple path mode control should be used for further processing. In a general case, the input state can be given as follows:


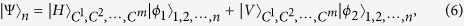


The control single photon could have *m* spatial modes (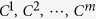), and the target photon could have *n* spatial modes (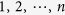). For other applications of such c-path gate, the input single photon states for a general *m*-control-*n* c-path gate can be directly prepared with linear optical circuits^62^,^63^. Through the similar procedure as in the previously discussed special c-path gate, one will obtain the following state before the detection:


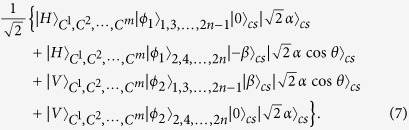


After the detection by the PND and the corresponding conditional phase shift if necessary, the following desired state can be achieved,


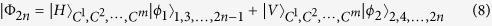


where the spatial modes 1, 3, 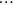, 2*n *− 1 of the target photon depend on the polarization (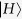) of the control photon; while the other spatial modes 2, 4, 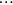, 2*n* depend on the polarization (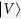) of the control photon.

In the above process, each spatial mode of the target qubit will be separated into two spatial modes, respectively, depending on the bit information of control qubit. Regarding the number of operations, all spatial modes of control and target single photon should interact with the qubus beams, necessitating 2*n* + 2*m* XPM processes in total. During an operation, the qubus beams are not destroyed, i.e. 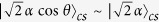, since *θ* is tiny. So they can be reused in the following operations. As discussed in the first part of Supplementary Material, these qubus beams can be used for more than 10^4^ times, even with a moderate strength, e.g. |α| ~ 10^3^, and a small cross phase shift, e.g. *θ* ~ 0.01. Moreover, one could reduce the number of XPM process to *n* + *m* by moving all interactions into one arm (see the second part of Supplementary Material for the details). Throughout an operation, only two coherent-state components should be consumed in detection. If *n* and *m* are very small, we may choose to save the qubus beams. The alternative is to lower the amount of XPM processes, given large *n* or *m*.

## Merging Gate

**Figure 2:**
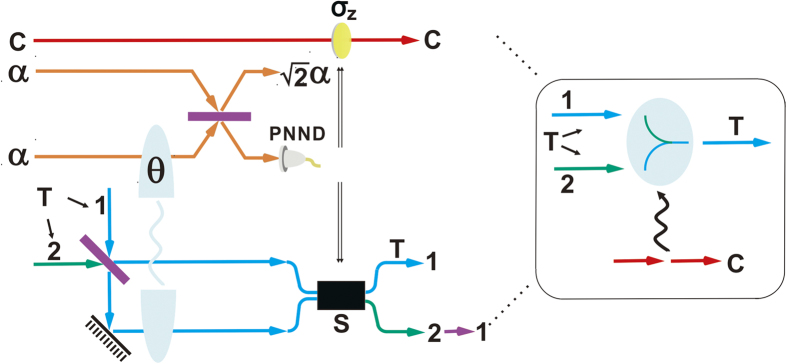
Schematic design of an example of merging gate. Firstly, the two spatial modes of the target photon undergo the interference via a 50:50 BS. The spatial mode 2 will interact with one of the coherent state. After that, the detection on the first coherent-state component is used to control the switch and the Pauli operation *σ*_*z*_, realizing the merging gate.

A merging gate performs the inverse transformation of a c-path gate. A special example of the gate for processing the quantum state from the input state in Eq. (4) is shown in Fig. 2. Firstly, the target photon with 2 spatial modes is injected into a 50:50 BS, and then the input state 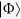 is transformed to


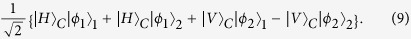


Letting the spatial mode 2 interact with the qubus beam as shown in Fig. 2, we will get the following state:
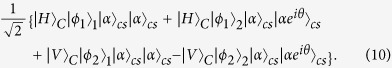
After that, one more 50:50 BS and the detection with the result *k* = 0 on the first qubus beam will project the above state into the desired state 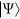. Meanwhile, the detection with the result 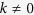 will project the state to 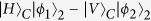, where the index 2 can be redefined as 1. Finally, one *σ*_*z*_ operation on the control photon and a switch of the upper and lower spatial modes, which are based on the classically feed-forwarded measurement, will transform the above state to the desired one 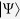.

Generalized to the case for more than one spatial mode of the control and target photon is straightforward. With the same setups, the initial state as the form of 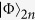 can be transformed back to the desired state as the form of 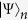. In one word, a merging gate depicted in the above merges the spatial modes of a target photon without changing anything else. Here, no ancilla single photon is required as compared with the design in the previous works^59^,^60^,^61^. Moreover, only the discrimination of the vacuum state 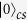 and the coherent state 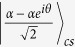 is necessary here, with their overlap being in the approximate order of exp{−*α*^2^*θ*^2^/4} to have the close to ideal discrimination of these two states. Similar to the operation of the PND module discussed in the first part of Supplementary Material, the discrimination of the vacuum and coherent state could be realized by a photon number non-resolving detector (PNND) with less than unit efficiency *η* < 1. The error probability of the operation is in the approximate order exp{−*ηα*^2^*θ*^2^/2}, demanding a moderate requirement 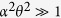. In contrast, the requirements in Homodyne detection scenarios are much tougher; 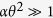 for the 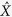-quadrature measurement^28^,^29^,^33^,^34^, and 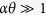 for the 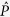-quadrature measurement^33^,^34^. Similar to the use of qubus beam in the PND module discussed in the first part of Supplementary Material, the remaining qubus beam is almost the same as the initial one 
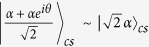
, so the qubus beam could be recycled for large number of times as well. Moreover, only half of the spatial modes of target photon should interact with the qubus beam, demanding only *n* XPM operations.

## Multi-control Unitary Operations and General Unitary Operation

Since the combination of a pair of c-path gate and merging gate (associated with a bit flip operation) can be used to realize a CNOT gate, these two element gates are universal for circuit-based quantum computation^59^. In addition, we will show that c-path and merging gate make it possible to realize various controlled unitary operations involving large number of qubits in more efficient way.

## General (*n*−1)-control-1 Unitary Operation

The first gate is a general (*n *− 1)-control-1 gate called uniform controlled rotation^11^ or multiplexor^14^. It implements an operation represented by the following matrix:


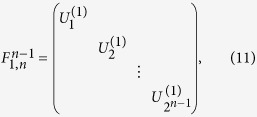


where the subscripts (1, *n*) means that the target qubit is the *n*-th qubit, and the superscript (*n *− 1) indicates that the control qubits are the other *n *− 1 qubits. 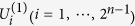 in the matrix are the single-qubit unitary operations. With 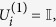 for 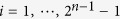, and 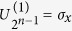, the gate is a 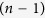-controlled Toffoli gate. To realize a Toffoli gate, 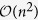 CNOT gates should be necessary^5^, while for a general (*n *− 1)-control-1 gate, the required CNOT gate number should be increased to 2^*n*−1^ 11. However, the complexity to realize a Toffoli gate can be reduced to linear by using *n *− 1 pairs of c-path and merging gates^59^,^60^. Here we will show that this approach can be generalized to realize a general (*n *− 1)-control-1 gate efficiently.

**Figure 3:**
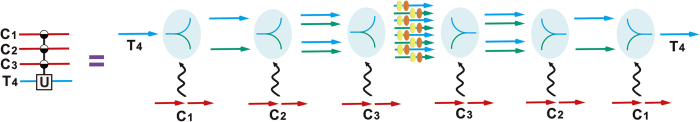
Realization of 3-control-1 unitary operation. By three c-path gates controlled by the photons *C*_1_, *C*_2_, *C*_3_ sequentially, the target photon will be separated into 8 spatial modes. After the single-photon unitary operations on the corresponding spatial modes, this unitary operation will be realized, associated with the operations of three merging gates.

For clarity, we use the example of 3-control-1 unitary operation for illustration (see Fig. 3). The input state can be the following general 4-qubit state, 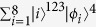, where 
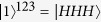
, 
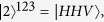
 etc. and 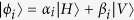 with 
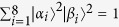
. As shown in Fig. 3, with the sequential operation of three c-path gates on the target photon, the input state will be transformed to 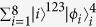, where the subscript *i* outside the bracket denotes the spatial modes of the fourth qubit. The eight spatial modes are determined by the bit information of the other three control qubits. If eight single-qubit unitary operations (
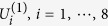
) are preformed on the corresponding spatial modes, one will obtain the state, 
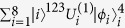
. Finally, after three merging gates erase the path information of the spatial modes *i*, the desired state 
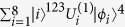
 will be achieved, realizing the 3-control-1 unitary operation. Generalizing to *n*-qubit case is straightforward with (*n *− 1) pairs of c-path and merging gates. It is significantly simpler than the traditional CNOT-based approach, which demands complicated decomposition into exponentially large number CNOT gates.

The quantity of XPM process for implementing the linearly scaling c-path and merging gate pairs is also an indicator for the complexity of the gate designs. By applying the c-path gates step by step, the involved spatial modes of the target photon will be exponentially increased with the operation procedure. Generally, for the *m*-th c-path gate, the target photon will be separated into 2^*m*^ spatial modes, which should be coupled to the qubus beam. Therefore, 2^*m*−1^ + 1 XPM processes are required in the *m*-th c-path gate (here the c-path gate is the modified one discussed in the second part of Supplementary Material). On the other hand, for the inverse *m*-th merging gate (the operation order is from *n *− 1 to 1), 2^*m*−1^ XPM processes are necessary too. Totally, the number of the necessary XPM process for the realization of a (*n *− 1)-control-1 gate should be


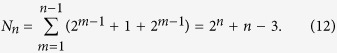


If the modified c-path gates are replaced by the original c-path gates discussed before, the required number of XPM process will be increased to
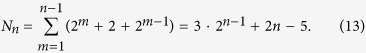
This exponential increasing is due to the fact that the general (*n *− 1)-control-1 gate itself is exponential complexity. Since there are exponential 2^*n*−1^ control unitary operations in such gate, the exponentially increasing number of XPM process will be inevitable.

In addition to the number of XPM process, the amount of other operations such as single photon interference, coherent state interference, as well as the required resources such as qubus beams (not including ancilla single photons), are only linearly increasing with the involved photonic qubit number. That is a considerable improvement over the former CNOT-based approach, which requires the amount of interferences, qubus beams, ancilla single photons and others in the exponential orders. In this sense, our current approach provides a more feasible way to realize a general (*n *− 1)-control-1 unitary operation.

## Special (*n*−1)-control-1 Unitary Operation

It is possible to optimize the implementation of the following special (*n *− 1)-control-1 gate operation


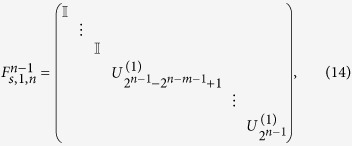


where *m *≤ *n *− 1. In this operation the target photon will not be affected when one of the first *m* control photons is in the state 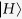, but it will be under operation when the first control photons are all in the state 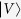.

**Figure 4:**
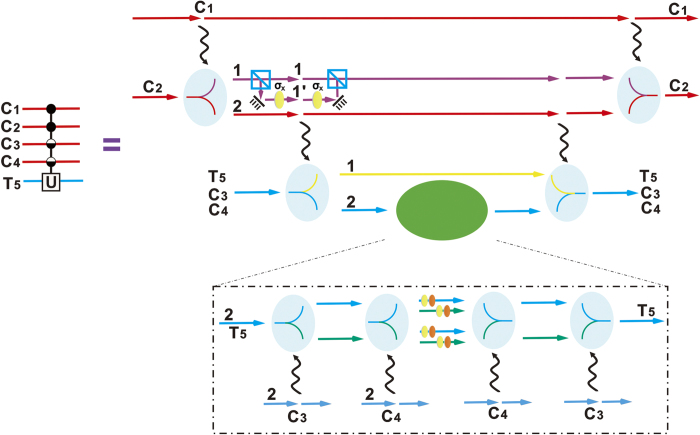
Procedure of implementing the 4-control-1 unitary operation outlined in the left panel. Step one: photon 1 controls photon 2 by the first c-path gate. Step two: the spatial mode 1 passes through a PBS and a *σ*_*x*_ operation to the 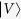 mode is applied. Step three: the three spatial modes are used as the control modes to control the other three photons 3, 4, 5 by three c-path gates. Final step: a general 2-control-1 operation to the spatial modes 2 of photons 3, 4, 5 is applied, together with the inverse merging gate operations.

Without loss of generality we use a 5-qubit gate in Fig. 4 as example. Here we briefly describe steps, and the details can be found in the third part of Supplementary Material. At first, the photon *C*_1_ will control the photon *C*_2_, but does not act on the target photon directly. Next, the three spatial modes (1, 1′, 2) of the photon 2 will control all rest photons, including the target photon. After that, the photon *C*_3_, *C*_4_, *T*_5_ will be separated into two spatial modes (1, 2), respectively. Applying the general 2-control-1 unitary operation on the spatial mode 2 of the photon *C*_3_, *C*_4_, *T*_5_, the desired 5-qubit gate will be completed associated with the corresponding merging gates.

Generalizing to the unitary operation of Eq. (14) is straightforward. Firstly, *m *− 1 c-path operations are performed to the first *m* photons in turn. After that, using the spatial modes (1, 1′, 2) of the *m*-th control photon as the control modes for the following *n *− *m* c-path gates, all of the rest photons including the target photon will be separated into two spatial modes (1, 2), respectively. Finally, by applying the general (*n *− *m *− 1)-control-1 unitary operation discussed before to the spatial modes 2 of the rest photons, associated with the corresponding *n *− 1 merging gates, the desired unitary operation will be completed.

Now we discuss the complexity of the procedure. The first modified c-path gate requires 2 XPM processes, and each of the other *n *− 2 modified c-path gates needs 2 XPM processes as well (the control photon has three spatial modes and two of them are in the state 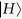, which will not interact with the qubus beam). For a general (*n *− *m *− 1)-control-1 gate, 2^*n*−*m*^ + *n *− *m *− 3 XPM processes are required. Meanwhile, for the *n *− 1 merging gate, *n *− 1 XPM processes are sufficient. The total number of XPM processes should be 2^*n*−*m*^ + 4*n *− *m *− 6. Compared with the amount of the general *n*-control-1 gate, it is a considerable improvement by reducing a factor of 2^*m*^. Obviously, it is on the same order of amount as the non-identity operations in Eq. (14). Especially, if *m* = *n *− 1, it will be a general Toffoli gate, which can be implemented with 3*n *− 3 XPM processes scaling linearly with *n*.

## 1-control-(*n*−1) Unitary Operation

**Figure 5:**
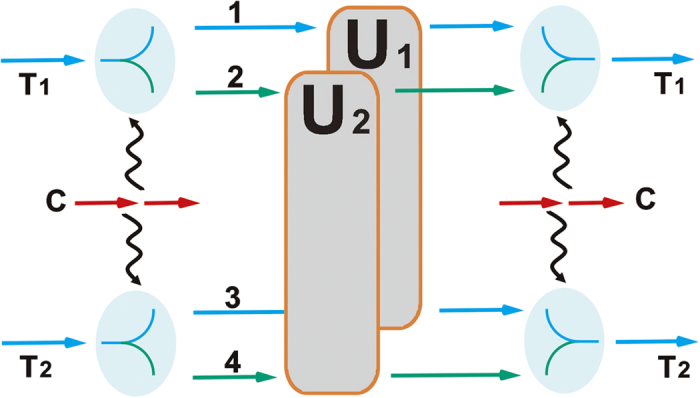
Implementation of 1-control-2 unitary operation. Firstly, apply two c-path gate operations between the control photon and the two target photons, respectively. After that, two unitary operations are applied to the corresponding spatial modes as shown in the figure. Finally, the whole procedure will be completed by two merging gates.

Now, we consider another *n*-qubit gate, through which one qubit controls the other *n *− 1 qubits. Its operation is described by the matrix


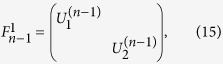


where 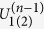 denotes a (*n *− 1)-qubit unitary operation. We first consider the example of 1-control-2 gate (see Fig. 5). This 1-control-2 unitary operation implements unitary operations 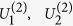 on two target qubits when the control qubit in the states 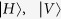, respectively. Suppose that the input state is 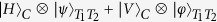, where 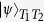 and 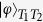 can be in arbitrary forms. Firstly, one uses two c-path gates to separate the two target qubits into two spatial modes (1, 2) or (3, 4), respectively, i.e. the obtained state is 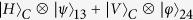. After that, implementing the desired unitary operations on the two spatial modes (2, 3) or (1, 4), respectively, will yield the following state


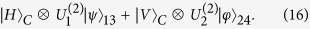


Finally, two merging gates are used to erase the path information, and then one will achieve the target state
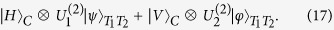
Here, two pairs of c-path and merging gate, associated with two two-qubit unitary operations, will be needed. The required sources are also obviously fewer than the CNOT-based approach. Especially, if 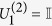 and 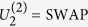, it will be a Fredkin gate. In the CNOT-based approach this gate requires 5 CNOT gates^6^ (one CNOT is equivalent to a pair of c-path and merging gate, or two parity-check operations^16^), while only two pairs of c-path and merging gates (associated with a spatial mode swap operation) are necessary to construct the gate^59^,^60^.

Its generalization is straightforward with three similar processes will complete the operation; (1) *n *− 1 c-path gates separate each of the *n *− 1 target qubits into two spatial modes; (2) the unitary operation 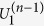 is performed on the spatial modes corresponding to the state 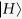 of the control qubit, and the unitary operation 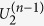 on the other spatial modes simultaneously; (3) *n *− 1 merging gates will merge the target qubits. Totally, except for the requirement for realizing unitary operations 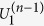 and 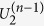, the required sources increase linearly with involved qubits number (*n *− 1 pairs of c-path and merging gates).

## *n*-control-*m* Unitary Operation

In what follows, we will discuss the *n*-control-*m* unitary operation, which is described by the following:


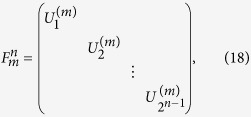


where 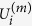 
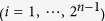
 are the *m*-qubit unitary operations. We combine the structures of the above gates to realize this *n*-control-*m* unitary operation. Firstly, we use *n* c-path gates to separate the first target qubit into 2^*n*−1^ spatial modes, and then use *n* c-path gates to separate the other target qubits, respectively. After that, we implement the unitary operation 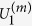 on the first spatial modes of each target qubits, followed by implementing the other *m*-qubit unitary operations 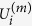 to the corresponding spatial modes. Finally, the merging gates will merge the target qubits to complete the whole procedure. Totally *n *× *m* pairs of c-path and merging gate are required, in addition to the *m*-qubit unitary operations.

## General *n*-qubit Unitary Operation

The most general operation is *n*-qubit unitary operation. This operation is a crucial operation in quantum information processing, since it simulate the evolution of *n* spin-1/2 interacting particles. This simulation is impossible by classical computer. A general n-qubit unitary operation has 4^*n*^ − 1 degrees of freedom from a 2^*n*^ × 2^*n*^ unitary matrix. Numerous works have been devoted to the problem of how to construct a general *n*-qubit unitary operation with two-qubit gates and single-qubit gates^4^,^7^,^8^,^12^,^13^,^14^. The theoretical lower bound of the CNOT approach is 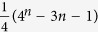
12. However, it is only a theoretical limit, and the detailed construction of such circuit had not discovered yet. The best circuit construction is the quantum Shannon decomposition (QSD), using (23/48)* *× 4^*n*^* *− (3/2)* *× 2^*n*^ + 4/3 CNOT gates^14^. Here we will present two approaches to the construction of a general *n*-qubit unitary operation with c-path and merging gates.

### Approach based on cosine-sine decomposition

The first approach is based on the cosine-sine decomposition (CSD)^68^,^69^. By this method a general *n*-qubit unitary operation can be decomposed into the following form:


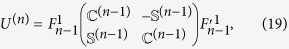


where 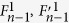 are the 1-control-(*n *− 1) unitary operations, and 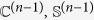 are the real diagonal matrices satisfying 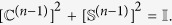 It has been demonstrated that the middle operation is equivalent to a (*n *− 1)-control-1 unitary operation^14^. With the above decomposition it is evident that one could combine two 1-control-(*n *− 1) unitary operations and one (*n *− 1)-control-1 unitary operation to realize a general *n*-qubit unitary operation. Therefore we will get the following recursive relation


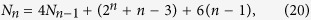


for the number of XPM process, where the second term is the amount of the middle (*n *− 1)-control-1 unitary operation, and the third is that of the c-path and merging gates used in the first turn of 1-control-(*n *− 1) gate. Then, the total number of XPM process is found as


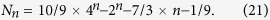


### Approach based on further decomposition into general (*n *− 1)-control-1 unitary operations

One can also decompose a general unitary operation without CSD. It was demonstrated that a general n-qubit unitary operation can be decomposed into a series of (*n *− 1)-control-1 unitary operations as follows^11^:


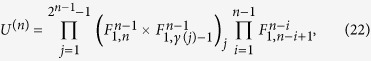


where the function *γ*(*j*) indicates the position of the least significant nonzero bit in the *n*-bit binary presentation of the number *j*. Obviously, the above decomposition allows the realization of general unitary operation, together with a general (*n *− 1)-control-1 unitary operation discussed before. There are 2(2^*n*−1^* *− 1) general (*n *− 1)-control-1 unitary operations (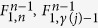) and (*n *− 1) general (*n *− *i*)-control-1 unitary operations (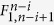). Therefore, the total number of the required XPM process will be


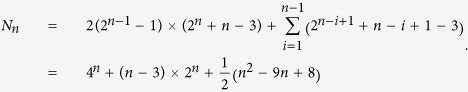


### Comparison between complexity

Now we compare our approaches with the CNOT-based approach in terms of their complexity. An optical CNOT gate demands two parity-check and one single photon as ancilla^16^. If assisted with weak cross-Kerr nonlinearity, two XPM processes will be needed for one parity-check^28^,^29^. This number can be reduced to one by saving one qubus beam at the price of lowering the success probability by half^48^. In other words, a CNOT gate requires two XPM processes, associated with one ancilla single photon in addition to the qubus beams. Alternatively one could use more XPM processes and qubus beams to have deterministic operation. In this case, a CNOT gate requires four XPM processes involving an ancilla single photon. Moreover, the number of interference processes should be taken into account. A parity-check operation works with one two-photon interference process and one coherent-state interference process, implying that a CNOT gate needs four interference processes.

In Table 1 we list the source requirements of the CNOT approach and our c-path-merging approach for comparison. There, each rows include two quantities, one for those using less XPM processes and more qubus beams, and the other for those using more XPM processes to save qubus beams that could be recycled. The first row is the theoretical lower bound of CNOT approach. Totally, 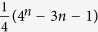 CNOT gates are required for a general unitary operation^12^. The quantities of XPM processes, qubus beams, ancilla single photons and interference processes are based on the number. The second row is about the CNOT-based circuit, with the optimal number (23/48)* *× 4^*n*^* *− (3/2)* *× 2^*n*^ + 4/3.

The required resources for our first approach based on CSD are given in the third row. The amount of XPM process for the modified c-path gate is shown in Eq. (21). Since the qubus beam will be detected with the probability 1/2, the corresponding amount of qubus beams can be calculated by the recursive relation *A*_*n*_ = 4*A*_*n*−1_ + 3(*n *− 1)/2. Exactly this is just half of the number of c-path-merging pairs used for the gate. Moreover, two interference processes are necessary in a c-path gate and a merging gate, respectively. Totally, 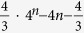 interference processes should be used in our first approach. If using the original c-path gate shown in Fig. 1, the amount of XPM processes will be increased to the scaling 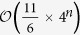, which is found by the relation


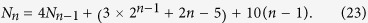


This is smaller than the corresponding number of the CNOT approach. Especially, in our approach, no ancilla single photon is necessary in contrast to the CNOT approach.

In addition to the amount of XPM processes shown in Tab. I, the corresponding number of qubus beams is
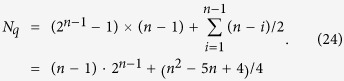
Obviously, this number scales as *n*2^*n*−1^, much lower than those of the three other approaches by a factor of 2^*n*^/*n*. Since this quantity happens to be half of the number of c-path-merging pairs, the number of required interference processes is eight times of this number. If using the original c-path gates, the required XPM processes will be increased to


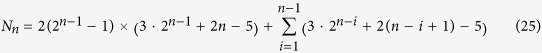


Through the comparison it is evident that our approaches enjoy the advantages of no ancilla single photons, less ancilla resources (qubus beams) and fewer operations (interference processes). Our second approach with the modified c-path gate is the optimal one to realize a general unitary operation. It should be noted that the sources for doing the measurements are not taken into account in the above discussion, since we only focus on the complexity of the schemes themselves and the measurements only use more sources of constant amount if they are performed by a few modules (like that described in the first part of Supplementary Material) in succession.

## Discussion on Feasibility of XPM

The crucial element in our approach is the XPM based on Kerr nonlinearity. Here we approximate the XPM as a single-mode process. In reality, however, photons carry continuous frequency distributions, and the multi-mode character can affect an XPM process. In view of the phase noise existing in non-instantaneous Kerr nonlinearity^70^, a multi-mode effect induced imperfection of XPM was first considered by Shapiro and collaborators^71^,^72^. They conclude that the phase noise due to the non-instantaneous response of Kerr medium can impair the ideal operation of XPM. The non-instantaneous response to light field can happen in optical fiber of silicon and other similar materials. With their extremely small Kerr coefficients, a considerably lengthy fiber should be used to generate a sufficient nonlinear phase. However, a dominant process in fiber is the absorption of the light, which leads to the decoherence of the generated photonic states^57^. This essential point excludes the feasibility of the setups that are relevant to the phase noise problem. On the other hand, the systems that realize much higher Kerr coefficients are the coherently prepared atomic ensembles under the conditions of electromagnetically induced transparency (EIT). The response of these atomic ensembles to the input light field is virtually immediate in activating the third and higher order nonlinearity as demonstrated by the experimental^73^,^74^,^75^ and theoretical studies^76^,^77^,^78^,^79^,^80^,^81^, thus neglecting the phase noise effect in such Kerr nonlinearities.

Another imperfections due to the multi-mode nature of inputs is the mode entanglement under photonic coupling or interaction^76^,^77^,^79^,^80^,^81^. Relevant to the Kerr nonlinearity based on atomic ensembles, this effect deviates a real XPM process from the ideal one 
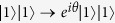
 for a pair of single photon states and 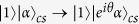 between a single photon and a coherent state. For the XPM considered in this paper, it is possible to eliminate the mode entanglement by adopting the counter-propagation configuration and transverse confinement of the inputs^79^, so that a close to single-mode XPM will be possible in a normal EIT medium. The improvement on the intensity of Kerr nonlinearity is feasible via the non-local atomic interaction in other atomic ensembles^81^. Currently both experimental and theoretical progress toward practical Kerr nonlinearity are under way.

## Conclusion

With the improved designs of c-path and merging gate, the realization of various control unitary operations can be more efficient and with less sources and operations. Compared with the widely considered approach based on CNOT gate and the previously proposed schemes based on the original c-path and merging gate, the improvement on the designs of some multi-qubit gates is significant. For example, a general *n*-control-1 gate can be realized by linearly increasing pairs of c-path and merging gate with the number of processed photons, while no ancilla photon is needed in operations. The close to ideal XPM process used in the circuits, as well as in the detection module, would be available with the development of the techniques of Kerr nonlinearity. Based on this prerequisite, the schemes proposed in the current study could become competitive alternatives for large scale photonic quantum computation with their considerably relaxed requirements on sources and operation times.

## Additional Information

**How to cite this article**: Lin, Q. and He, B. Highly Efficient Processing of Multi-photon States. *Sci. Rep.* **5**, 12792; doi: 10.1038/srep12792 (2015).

## Supplementary Material

Supplementary Information

## Figures and Tables

**Table 1:** Comparison of CNOT approach and c-path-merging approach.

	XPM processes	qubus beams	ancilla single photons	interference processes
CNOT-1^12^	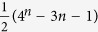 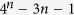	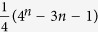	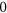 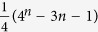	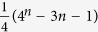 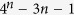
CNOT-2^14^	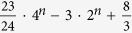 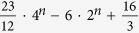	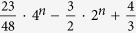 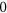	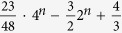 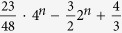	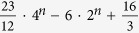
c-path-merging-1	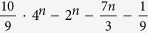 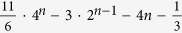	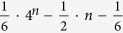 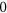	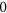 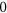	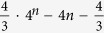
c-path-merging-2	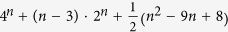 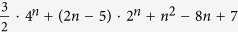	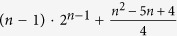 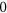	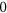 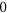	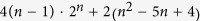

The first and second rows are the theoretical lower bound and the known-circuit of CNOT-based approach, respectively, while the third and fourth rows are for the two c-path-merging approaches based on CSD directly and further decomposition, respectively.
