# Supplementary material for: Highly Efficient Processing of Multi-photon States
Source: Sci Rep. 2015 Aug 6;5:12792. doi: 10.1038/srep12792 (PMC4526856; doi:10.1038/srep12792)
Supplement: Supplementary Information [file srep12792-s1.pdf]

Supplementary Material for "Highly Efficient Processing Multi-photon States"

Qing Lin*
Fujian Provincial Key Laboratory of Light Propagation and Transformation,
College of Information Science and Engineering, Huaqiao University, Xiamen 361021, China

Bing He†
Department of Physics, University of Arkansas, Fayetteville, AR 72701, USA

The photon number-resolving detection module

We discuss the realization of the photon number-resolving detector (PND) in this part. The idea of our design is to realize the PND in an indirect way with photon number non-resolving detector (of less than unit efficiency) and coherent state comparison. More details of the proposal can be found in [1, 2]. Here we present an outline of the implementation of the PND module with Fig. A-1. Suppose that the photon number non-resolving detector used in the module has the efficiency $\eta < 1$. The aim is to resolve the exact Fock state components in the coherent state component $|\pm\beta\rangle_{cs} = \left|\pm i\sqrt{2}\alpha\sin\theta\right\rangle_{cs}$ with $\theta \ll 1$. We let it interacted with one of the two qubus beams $|\gamma\rangle_{cs}|\gamma\rangle_{cs}$ through an XPM process. The transformation of the qubus beam state is as follows,

$$|\pm\beta\rangle_{cs}|\gamma\rangle_{cs}|\gamma\rangle_{cs} \to e^{-|\beta|^2/2}\sum_{k=0}^{\infty}\frac{(\pm\beta)^k}{\sqrt{k!}}|k\rangle\left|\gamma e^{ik\theta}\right\rangle_{cs}|\gamma\rangle_{cs}. \tag{A-1}$$

After that, one more beam splitter (BS) is applied to get the state

$$e^{-|\beta|^2/2}\sum_{k=0}^{\infty}\frac{(\pm\beta)^k}{\sqrt{k!}}|k\rangle\left|\frac{\gamma e^{ik\theta}-\gamma}{\sqrt{2}}\right\rangle_{cs}\left|\frac{\gamma e^{ik\theta}+\gamma}{\sqrt{2}}\right\rangle_{cs}. \tag{A-2}$$

Now the information of the Fock state components in $|\pm\beta\rangle_{cs}$ has been contained in the coherent state $\left|\frac{\gamma e^{ik\theta}-\gamma}{\sqrt{2}}\right\rangle_{cs}$.

For the different numbers k, the Poisson distributions of the coherent states $\left|\frac{\gamma e^{ik\theta}-\gamma}{\sqrt{2}}\right\rangle_{cs}$, for $k = 1, 2, \ldots,$ can be well separated with the approximate overlap $\exp\{-\gamma^2\theta^2/4\}$, given a sufficiently large $|\gamma|$; see Fig. A-1. It makes the discrimination of the coherent states $\left|\frac{\gamma e^{ik\theta}-\gamma}{\sqrt{2}}\right\rangle_{cs}$ for the different k possible. The response of a photon number non-solving detector to these coherent states, which occur with the corresponding probabilities as in the above equation, will be distinct. Moreover, due to the the small $|\beta|$, the number of the Poisson peaks in Fig. A-1 is limited. Thus, the projection $|k\rangle\langle k|$ on the coherent $|\pm\beta\rangle_{cs}$ will be realized indirectly with the module in Fig. A-1.

The operation of the photon number non-resolving detector can be described by the following positive operator valued measure (POVM) elements [3],

$$\begin{aligned}\Pi_0 &= \sum_{k=0}^{\infty}(1-\eta)^k|k\rangle\langle k|,\\ \Pi_1 &= I - \Pi_0,\end{aligned} \tag{A-3}$$

where Π_0 and Π_1 represent the detections of no photon or any number of photon, respectively. For the separated Poisson peaks in Fig. A-1, the second POVM element is effectively decomposed into

$$\Pi_1 = \Pi_1^{(1)} + \Pi_1^{(2)} + \cdots + \Pi_1^{(m)} + \cdots, \tag{A-4}$$

*Electronic address: qlin@mail.ustc.edu.cn
†Electronic address: binghe@uark.edu

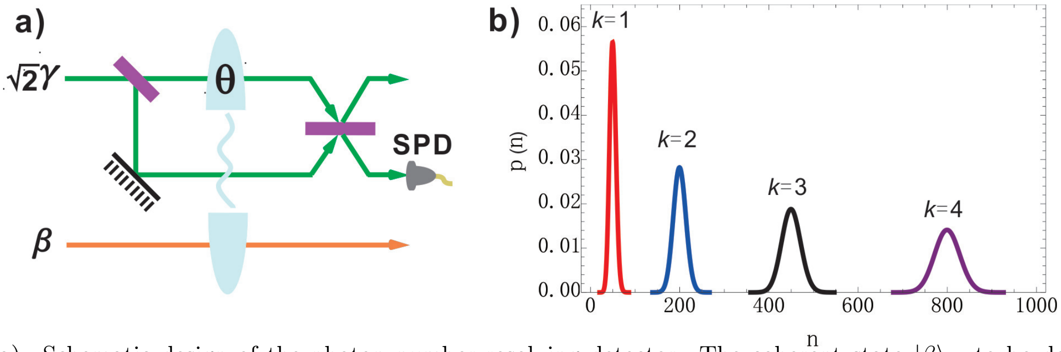

FIG. A-1: a). Schematic design of the photon number-resolving detector. The coherent state $|\beta\rangle_{cs}$ to be detected interacts with one of the qubus beams $|\gamma\rangle_{cs}|\gamma\rangle_{cs}$. After that, the two qubus beams interfere on a 50:50 BS. Finally, one of the qubus beams is detected by photon number non-resolving detector. b) The Poisson distributions of the coherent states $\left|\frac{\gamma e^{ik\theta}-\gamma}{\sqrt{2}}\right\rangle_{cs}$ for $k=1,2,3,4$. The amplitude of qubus beam is $|\gamma|=10^3$ and the cross phase shift is $\theta=0.01$.

where $\Pi_1^{(m)}=\sum_{k=m_i}^{m_f}\left(1-(1-\eta)^k\right)|k\rangle\langle k|$ with the ranges $[m_i,m_f]$ being not overlapped, and the m distinct readings of the detector corresponds the non-zero Poisson peaks of limited number. Meanwhile, the error probability of detecting nothing is

$$\begin{aligned}P_E &= \left\|\sum_{k=0}^{\infty} e^{-|\beta|^2/2}\frac{(\pm\beta)^k}{\sqrt{k!}}|k\rangle\,\Pi_0^{1/2}\left|\frac{\gamma e^{ik\theta}-\gamma}{\sqrt{2}}\right\rangle_{cs}\right\|^2\\ &\sim \exp\{-2(1-e^{-\eta\gamma^2\theta^2/2})\alpha^2\sin^2\theta\}.\end{aligned} \tag{A-5}$$

Evidently, given the parameters $\eta\gamma^2\theta^2\gg 1$ and $\alpha^2\sin^2\theta\gg 1$, the PND can be ideally performed with weak nonlinearity ($\theta\ll 1$).

The conditions on the ideal performance is different from the methods of Homodyne detection. If using $\hat{X}$-quadrature measurement, the requirement for deterministic operation is $\alpha\theta^2\gg 1$ [4–7]. The strength of coherent state must be much larger under the condition $\theta\ll 1$. If $\hat{P}$-quadrature measurement is used, the requirement could be improved to $\alpha\theta\gg 1$, but the operation will be probabilistic with the success probability 1/2 [6, 7]. As a comparison, the requirement for our design is only $\alpha^2\sin^2\theta\gg 1$. Moreover, the input coherent states can be used recycled. For example, given $\theta=0.01$ and $\alpha=10^3$, the corresponding average photon number of input qubus beam is $2|\alpha|^2=2\times 10^6$, and that of the detected coherent state is about $|\beta|^2\sim 200$. While ensuring the negligible error probability P_E calculated with Eq. (A-5), such qubus beams can be used for many times, because only small portion of photons is consumed in each detection as compared with the average number of photons carried by the qubus beams themselves. With the qubus beam $|\alpha\cos^t(\theta)\rangle_{cs}$ and $\gamma=10^2$ after use of $t=10^4$ times, the error probability P_E is still lower than 10^{-8}. The similar setting is also valid to the qubus beams $|\sqrt{2}\gamma\rangle_{cs}$ in the PND module.

Simplification of c-path gate

The realization of the general c-path gate can be simplified by removing all XPM processes on one arm as in Fig. B-1. After the XPM processes are applied, the initial state

$$\begin{aligned}|\Psi\rangle_n &= |H\rangle_{C^1,C^2,\cdots,C^m}|\phi_1\rangle_{1,2,\cdots,n}\\ &\quad+|V\rangle_{C^1,C^2,\cdots,C^m}|\phi_2\rangle_{1,2,\cdots,n},\end{aligned} \tag{B-1}$$

will be transformed to

$$\begin{aligned}&\frac{1}{\sqrt{2}}\Big\{|H\rangle_{C^1,C^2,\cdots,C^m}|\phi_1\rangle_{1,3,\ldots,2n-1}\left|\alpha e^{i\theta}\right\rangle_{cs}|\alpha\rangle_{cs}\\ &+|H\rangle_{C^1,C^2,\cdots,C^m}|\phi_1\rangle_{2,4,\ldots,2n}|\alpha\rangle_{cs}|\alpha\rangle_{cs}\\ &+|V\rangle_{C^1,C^2,\cdots,C^m}|\phi_2\rangle_{1,3,\ldots,2n-1}\left|\alpha e^{i2\theta}\right\rangle_{cs}|\alpha\rangle_{cs}\\ &+|V\rangle_{C^1,C^2,\cdots,C^m}|\phi_2\rangle_{2,4,\ldots,2n}\left|\alpha e^{i\theta}\right\rangle_{cs}|\alpha\rangle_{cs}\Big\}.\end{aligned} \tag{B-2}$$

After a phase shifter of $-\theta$ is applied, and the two qubus beams are interfered on a BS, the above state will be transformed to

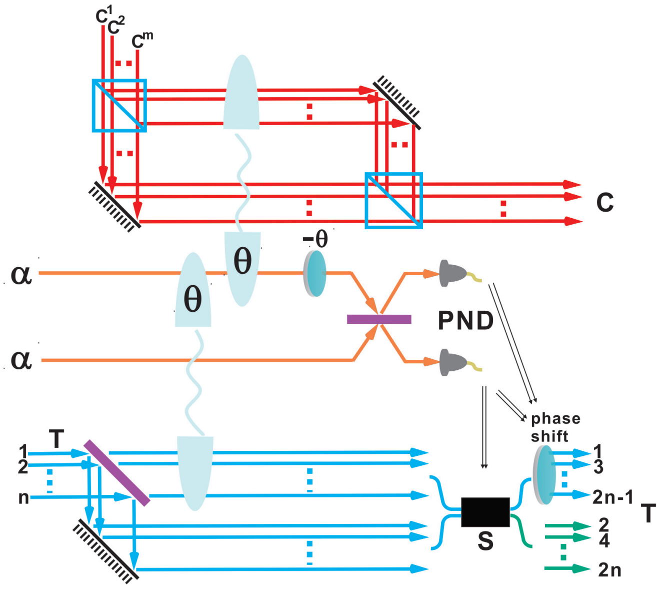

FIG. B-1: Schematic diagram of the modified general controlled-path gate. Compared with the original special c-path gate provided in Fig.1, the XPM processes on the second coherent state are removed totally. Moreover, two coherent-state components will be detected by the PNDs. In this realization, the coherent states cannot be recycled, but only half amount of XPM processes is necessary.

$$\begin{aligned}&\frac{1}{\sqrt{2}}\Big\{|H\rangle_{C^1,C^2,\cdots,C^m}|\phi_1\rangle_{1,3,\ldots,2n-1}|0\rangle_{cs}\left|\sqrt{2}\alpha\right\rangle_{cs}\\&+|H\rangle_{C^1,C^2,\cdots,C^m}|\phi_1\rangle_{2,4,\ldots,2n}\left|\frac{\alpha e^{-i\theta}-\alpha}{\sqrt{2}}\right\rangle_{cs}\left|\frac{\alpha e^{-i\theta}+\alpha}{\sqrt{2}}\right\rangle_{cs}\\&+|V\rangle_{C^1,C^2,\cdots,C^m}|\phi_2\rangle_{1,3,\ldots,2n-1}\left|\frac{\alpha e^{i\theta}-\alpha}{\sqrt{2}}\right\rangle_{cs}\left|\frac{\alpha e^{i\theta}+\alpha}{\sqrt{2}}\right\rangle_{cs}\\&+|V\rangle_{C^1,C^2,\cdots,C^m}|\phi_2\rangle_{2,4,\ldots,2n}|0\rangle_{cs}\left|\sqrt{2}\alpha\right\rangle_{cs}\Big\},\end{aligned}\tag{B-3}$$

where $\left|\frac{\alpha e^{\mp i\theta}-\alpha}{\sqrt{2}}\right\rangle_{cs}=\left|\pm i\sqrt{2}\alpha\sin(\theta/2)\,e^{\pm i\theta/2}\right\rangle_{cs}\simeq\left|\pm i\alpha\theta/\sqrt{2}e^{\pm i\theta/2}\right\rangle_{cs}$, $\left|\frac{\alpha e^{\pm i\theta}+\alpha}{\sqrt{2}}\right\rangle_{cs}=\left|\sqrt{2}\alpha\cos(\theta/2)\,e^{\pm i\theta/2}\right\rangle_{cs}\simeq\left|\sqrt{2}\alpha e^{\pm i\theta/2}\right\rangle_{cs}$. By the projection $|k\rangle\langle k|$ on the first qubus beam, the target state $|\Phi\rangle_{2n}$ can be obtained with the condition $k=0$. In this case, the qubus beam could be recycled. If $k\neq 0$, we will get the following state,

$$\begin{aligned}&e^{-i(k\pi-k\theta)/2}|H\rangle_{C^1,C^2,\cdots,C^m}|\phi\rangle_{2,4,\ldots,2n}\left|\sqrt{2}\alpha e^{-i\theta/2}\right\rangle_{cs}\\&+e^{i(k\pi-k\theta)/2}|V\rangle_{C^1,C^2,\cdots,C^m}|\psi\rangle_{1,3,\ldots,2n-1}\left|\sqrt{2}\alpha e^{i\theta/2}\right\rangle_{cs}.\end{aligned}\tag{B-4}$$

Since the coherent-state component in the two terms are different, one should measure the second qubus beam, *i.e.*, the qubus beam will be lost. If the result is l, the above state will be projected to

$$\begin{aligned}&e^{-i(k\pi-k\theta+l\theta)/2}|H\rangle_{C^1,C^2,\cdots,C^m}|\phi\rangle_{2,4,\ldots,2n}\left|\sqrt{2}\alpha e^{-i\theta/2}\right\rangle_{cs}\\&+e^{i(k\pi-k\theta+l\theta)/2}|V\rangle_{C^1,C^2,\cdots,C^m}|\psi\rangle_{1,3,\ldots,2n-1}\left|\sqrt{2}\alpha e^{i\theta/2}\right\rangle_{cs}.\end{aligned}\tag{B-5}$$

Since the exact values of k and l are known, the unwanted phase factor could be removed. Therefore the above state can be transformed to the desired state $|\Phi\rangle_{2n}$ through classical feedforward. Compared with the c-path in Fig.1, the amount of XPM processes could be reduced to $n+m$. The cost is the qubus beam (with the probability 1/2 when the first detection $k\neq 0$) and one more projection $|l\rangle\langle l|$. If n and m is very small, one may choose to save the coherent state. If n or m is large, we may choose to reduce the amount of XPM processes.

The procedure of realizing the special 4 control 1 gate in Fig.4

The operations shown in Fig.4 are used to realize the following 4-control-1 gate:

$$F_{s,1,5}^{4}=\begin{pmatrix} \mathbb{I} & & & & & \\ & \vdots & & & & \\ & & \mathbb{I} & & & \\ & & & U_{13} & & \\ & & & & \vdots & \\ & & & & & U_{16} \end{pmatrix}. \tag{C-1}$$

Obviously, the single qubit operations $U_{13},\ldots,U_{16}$ will be implemented to the target photon only when the first two photons are all in the state $|V\rangle$. The initial state can be described as follows:

$$\begin{aligned} &|HHHH\rangle|\phi_1\rangle+|HHHV\rangle|\phi_2\rangle+|HHVH\rangle|\phi_3\rangle\\ &+|HHVV\rangle|\phi_4\rangle+|HVHH\rangle|\phi_5\rangle+|HVHV\rangle|\phi_6\rangle\\ &+|HVVH\rangle|\phi_7\rangle+|HVVV\rangle|\phi_8\rangle+|VHHH\rangle|\phi_9\rangle\\ &+|VHHV\rangle|\phi_{10}\rangle+|VHVH\rangle|\phi_{11}\rangle+|VHVV\rangle|\phi_{12}\rangle\\ &+|VVHH\rangle|\phi_{13}\rangle+|VVHV\rangle|\phi_{14}\rangle+|VVVH\rangle|\phi_{15}\rangle\\ &+|VVVV\rangle|\phi_{16}\rangle, \end{aligned} \tag{C-2}$$

where $|\phi_i\rangle=\alpha_i|H\rangle+\beta_i|V\rangle$, $\sum_{i=1}^{16}\left(|\alpha_i|^2+|\beta_i|^2\right)=1$. First, let the first photon controls the second photon through the first c-path gate. After that, let the first spatial mode 1 of the second photon passed through a PBS and perform a σ_x operation on the $1'$ mode, yielding the following state

$$\begin{aligned} &(|HHHH\rangle|\phi_1\rangle+|HHHV\rangle|\phi_2\rangle+|HHVH\rangle|\phi_3\rangle\\ &+|HHVV\rangle|\phi_4\rangle)_1+(|HHHH\rangle|\phi_5\rangle+|HHHV\rangle|\phi_6\rangle\\ &+|HHVH\rangle|\phi_7\rangle+|HHVV\rangle|\phi_8\rangle)_{1'}+(|VHHH\rangle|\phi_9\rangle\\ &+|VHHV\rangle|\phi_{10}\rangle+|VHVH\rangle|\phi_{11}\rangle+|VHVV\rangle|\phi_{12}\rangle\\ &+|VVHH\rangle|\phi_{13}\rangle+|VVHV\rangle|\phi_{14}\rangle+|VVVH\rangle|\phi_{15}\rangle\\ &+|VVVV\rangle|\phi_{16}\rangle)_2, \end{aligned} \tag{C-3}$$

where the subscripts outside the bracket denote the spatial modes of the second photon. Second, using the three spatial modes of the second photon to control the photon $3,4,5$ by three c-path gates, one will obtain the following state

$$\begin{aligned} &(|HHHH\rangle|\phi_1\rangle+|HHHV\rangle|\phi_2\rangle+|HHVH\rangle|\phi_3\rangle\\ &+|HHVV\rangle|\phi_4\rangle)_{1111}+(|HHHH\rangle|\phi_5\rangle+|HHHV\rangle|\phi_6\rangle\\ &+|HHVH\rangle|\phi_7\rangle+|HHVV\rangle|\phi_8\rangle)_{1'111}+(|VHHH\rangle|\phi_9\rangle\\ &+|VHHV\rangle|\phi_{10}\rangle+|VHVH\rangle|\phi_{11}\rangle+|VHVV\rangle|\phi_{12}\rangle)_{2111}\\ &+(|VVHH\rangle|\phi_{13}\rangle+|VVHV\rangle|\phi_{14}\rangle+|VVVH\rangle|\phi_{15}\rangle\\ &+|VVVV\rangle|\phi_{16}\rangle)_{2222}, \end{aligned} \tag{C-4}$$

where the subscripts outside the bracket denote the spatial modes of the photons except for the first one. Obviously, the photons $3,4,5$ will be separated into the spatial mode 2 only when the first two photons are all in the state $|V\rangle$. Therefore, following the processes in the part of "general $(n-1)$-control-1 unitary operation" to operate on all the

spatial modes 2 of the photons $3, 4, 5$, one will obtain the state

$$
\begin{aligned}
&(|HHHH\rangle|\phi_1\rangle + |HHHV\rangle|\phi_2\rangle + |HHVH\rangle|\phi_3\rangle \\
&+|HHVV\rangle|\phi_4\rangle)_{1111} + (|HHHH\rangle|\phi_5\rangle + |HHHV\rangle|\phi_6\rangle \\
&+|HHVH\rangle|\phi_7\rangle + |HHVV\rangle|\phi_8\rangle)_{1'111} + (|VHHH\rangle|\phi_9\rangle \\
&+|VHHV\rangle|\phi_{10}\rangle + |VHVH\rangle|\phi_{11}\rangle + |VHVV\rangle|\phi_{12}\rangle)_{2111} \\
&+(|VVHH\rangle \otimes U_{13}|\phi_{13}\rangle + |VVHV\rangle \otimes U_{14}|\phi_{14}\rangle \\
&+|VVVH\rangle \otimes U_{15}|\phi_{15}\rangle + |VVVV\rangle \otimes U_{16}|\phi_{16}\rangle)_{2222}\,.
\end{aligned}
\tag{C-5}
$$

Finally, the inverse merging gates are applied to the corresponding single photons to transform the above state to the target state

$$
\begin{aligned}
&|HHHH\rangle|\phi_1\rangle + |HHHV\rangle|\phi_2\rangle + |HHVH\rangle|\phi_3\rangle \\
&+|HHVV\rangle|\phi_4\rangle + |HHHH\rangle|\phi_5\rangle + |HHHV\rangle|\phi_6\rangle \\
&+|HHVH\rangle|\phi_7\rangle + |HHVV\rangle|\phi_8\rangle + |VHHH\rangle|\phi_9\rangle \\
&+|VHHV\rangle|\phi_{10}\rangle + |VHVH\rangle|\phi_{11}\rangle + |VHVV\rangle|\phi_{12}\rangle \\
&+|VVHH\rangle \otimes U_{13}|\phi_{13}\rangle + |VVHV\rangle \otimes U_{14}|\phi_{14}\rangle \\
&+|VVVH\rangle \otimes U_{15}|\phi_{15}\rangle + |VVVV\rangle \otimes U_{16}|\phi_{16}\rangle\,.
\end{aligned}
\tag{C-6}
$$

This is how a special 4-control-1 gate is implemented.

---

[1] Lin, Q. & He, B. Single-photon logic gates using minimal resources. *Phys. Rev. A* **80**, 042310 (2009).

[2] Lin, Q. He, B. Bergou, J. A. & Ren, Y. H. Processing multiphoton states through operation on a single photon: Methods and applications. *Phys. Rev. A* **80**, 042311 (2009).

[3] Kok, P. & Braunstein, S. L. Postselected versus nonpostselected quantum teleportation using parametric down-conversion. *Phys. Rev. A* **61**, 042304 (2000).

[4] Barrett, S. D. et al. Symmetry analyzer for nondestructive Bell-state detection using weak nonlinearities. *Phys. Rev. A* **71**, 060302 (R) (2005).

[5] Nemoto, K. & Munro, W. J. Nearly Deterministic Linear Optical Controlled-NOT Gate. *Phys. Rev. Lett.* **93**, 250502 (2004).

[6] Louis, S. G. R. Nemoto, K. Munro, W. J. & Spiller, T. P. Weak nonlinearities and cluster states. *Phys. Rev. A* **75**, 042323 (2007).

[7] Louis, S. G. R. Nemoto, K. Munro, W. J. & Spiller, T. P. The efficiencies of generating cluster states with weak nonlinearities. *New J. Phys.* **9**, 193 (2007).
